# Unravelling mycorrhiza-induced wheat susceptibility to the English grain aphid *Sitobion avenae*

**DOI:** 10.1038/srep46497

**Published:** 2017-04-13

**Authors:** Amma L. Simon, Peter A. D. Wellham, Gudbjorg I. Aradottir, Alan C. Gange

**Affiliations:** 1 Department of Biological Chemistry and Crop Protection, Rothamsted Research, Harpenden, Hertfordshire, AL5 2JQ, UK; 2 School of Biological Sciences, Royal Holloway University of London, Egham, Surrey, TW20 0EX, UK; 3 Somerville College, University of Oxford, Oxford, OX2 5HD, UK

## Abstract

Arbuscular mycorrhizal (AM) fungi are root symbionts that can increase or decrease aphid growth rates and reproduction, but the reason by which this happens is unknown. To investigate the underlying mechanisms of this interaction, we examined the effect of AM fungi on the English Grain aphid (*Sitobion avenae*) development, reproduction, attraction, settlement and feeding behaviour on two naturally susceptible varieties *Triticum aestivum* (L.) variety Solstice and *T. monococcum* MDR037, and two naturally resistant lines, *T. monococcum* MDR045 and MDR049. Mycorrhizal colonisation increased the attractiveness of *T. aestivum* var. Solstice to aphids, but there was no effect on aphid development on this variety. Using the Electrical Penetration Graph (EPG) technique, we found that mycorrhizal colonisation increased aphid phloem feeding on *T. monococcum* MDR037 and MDR045, colonisation also increased growth rate and reproductive success of *S. avenae* on these varieties. Mycorrhizas increased vascular bundle size, demonstrating that these fungi can influence plant anatomy. We discuss if and how this could be related to an enhanced success rate in phloem feeding in two varieties. Overall, we present and discuss how mycorrhizal fungi can affect the feeding behaviour of *S. avenae* in wheat, inducing susceptibility in a resistant variety.

Aphids belong to the superfamily Aphidoidea which is thought to have evolved about 280 million years ago, in the Permian period, although the oldest fossil aphid is from the Triassic. Aphid diversification occurred simultaneously with the diversification of Angiosperms in the early Cretaceous^1^. Most of the ~4700 aphid species^2^ are phloem feeders and have developed methods of bypassing the plant defences so that they can remain at a feeding site for days^3^.

Plants have complex systems of defence in response to herbivores, with few, if any of the underlying traits acting independently^4^,^5^. Studies have shown that resistance traits are under selection from herbivores^4^,^6^,^7^,^8^,^9^. For this to happen there must be additive genetic variation in resistance traits^10^. Selection agents for this resistance are unclear, but both plant defence theory and plant–herbivore coevolutionary theory assume that resistance traits have evolved as adaptations to reduce herbivory^10^. Comparative studies on a set of closely related species or plant varieties could hold the key to identifying traits for plant defence and aphid performance^9^. However, it is not just plant traits that determine insect resistance, but the interaction with other organisms, such as fungi, and therefore such studies need to be set in a multitrophic context^11^.

It has long been known that agricultural intensification and homogeneity causes increased herbivorous insect infestations and increases the selection pressure on sap-feeding insects to overcome plant resistance^12^. Renewed interest in understanding aphid behaviour and host preferences are largely due to the recent development of insecticide resistance in many aphid species^13^ which, coupled with increasing restrictions on insecticide use^14^, are causing serious concerns for food security. Wheat is a crop of high economic importance as it is a main ingredient of many diets around the world^15^,^16^. The English grain aphid *Sitobion avenae* (F.) commonly infests wheat and other cereal crops across Europe, the Americas and Asia. This aphid can reduce yield by feeding on phloem sap which removes nutrients, secreting sucrose-rich honeydew onto leaves thereby attracting fungi that reduce the plants’ photosynthetic ability, and lastly by transmitting plant viruses, most notably in the case of wheat, the Barley Yellow Dwarf Virus^17^.

Currently the main measure used to control pest populations are insecticides, however; *S. avenae* populations have developed resistance to classes of pesticides rendering them ineffective^13^, resulting in widespread infestation^18^ and focusing efforts on finding alternative control methods.

No natural resistance to *S. avenae* has been recorded in modern commercial cultivars of wheat; however, some ancestral varieties have shown resistance to aphid species^19^. *Triticum monococcum* (L.) is a diploid wheat cultivar that was grown as an agricultural crop in the Neolithic Age until the Bronze Age^20^. Some varieties have been shown to possess at least partial resistance to aphid species including *Rhopalosiphum padi* (L.)^21^,^22^ and *S. avenae*^23^.

Arbuscular mycorrhizal (AM) fungi create a symbiotic relationship with many plants^24^ by increasing the acquisition of nutrients, namely nitrogen and phosphorus, in exchange for carbon. In a meta-analysis, Koricheva *et al*.^25^ showed that mycorrhizas have an overall positive effect on phloem feeders; however, the effect of AM fungi on invertebrate herbivores depends on many biotic factors, most notably the host species^26^. On the one hand, mycorrhizas have been shown to increase aphid growth rates^27^,^28^ and change the Volatile Organic Compounds (VOCs) produced, increasing the attractiveness of un-infested plants to aphids^28^. On the other hand, AM fungi can change plant chemistry^29^ to alter the available resources within the plant causing a decrease in aphid populations^30^. There is also one brief report of AM fungi reducing aphid growth rates on wheat^31^, but the mechanism was not determined. Finally, there are also reports of AM fungi having no effect on aphid performance^32^.

The mechanisms by which AM fungi influence aphid growth and life history traits are largely unknown. Aphids are often responsive to changes in amino acid content of phloem sap, but when such changes have been searched for in mycorrhizal experiments, they have been absent^27^,^33^,^34^. Instead, it has been suggested that it is more likely due to mycorrhizal-induced increases in leaf vascular bundle size^35^, leading to increased phloem feeding. In resistant varieties of wheat, the mechanism is thought to be linked to the phloem, based on increased salivation and an inability by aphids to carry out sustained feeding^22^. Thus, to understand the mechanism of AM-aphid interactions, it is instructive to compare aphid performance on mycorrhizal and non-mycorrhizal varieties that differ naturally in resistance.

Here, we investigate the interaction between arbuscular mycorrhizal fungi, *S. avenae* and the diploid *T. monococcum* aphid susceptible variety MDR037, aphid resistant varieties MDR045 and MDR049 and the commercial hexaploid *T. aestivum* variety Solstice. These lines were identified in previous work to have varying levels of susceptibility to *R. padi*^22^. We determined whether mycorrhizal colonisation can affect the resistance and susceptibility that diploid *T. monococcum* varieties, and *T. aestivum*, hold towards *S. avenae*, through fecundity and development studies using whole plant cages.

This study also investigated if resistance in *T. monococcum* varieties was due to antixenosis and/or antibiosis and if this resistance can be affected by AM fungi. Antixenosis resistance was measured through attraction and settlement bioassays using winged (alate) *S. avenae*; these alates are produced when overcrowding occurs in order to find another host. Antibiosis resistance was measured using fecundity and development studies. To discover the mechanism underlying antibiosis resistance, the leaf vascular bundle size was measured, and *S. avenae* feeding behaviour was investigated using the Electrical Penetration Graph (EPG) technique^36^. To our knowledge, no study to date has used EPG to compare aphid feeding on mycorrhizal and non-mycorrhizal plants.

We investigated four hypotheses: 1) AM fungi increase the development and fecundity of *S. avenae*, 2) mycorrhizal colonisation increases the attractiveness of *T. aestivum* var. Solstice and *T. monococcum* varieties resulting in more aphids settling on treated plants, 3) that AM fungi influences vascular bundle size, and 4) AM fungi influence feeding behaviour, through increased sap ingestion.

## Results

### Mycorrhizal colonisation

**Figure 1:**
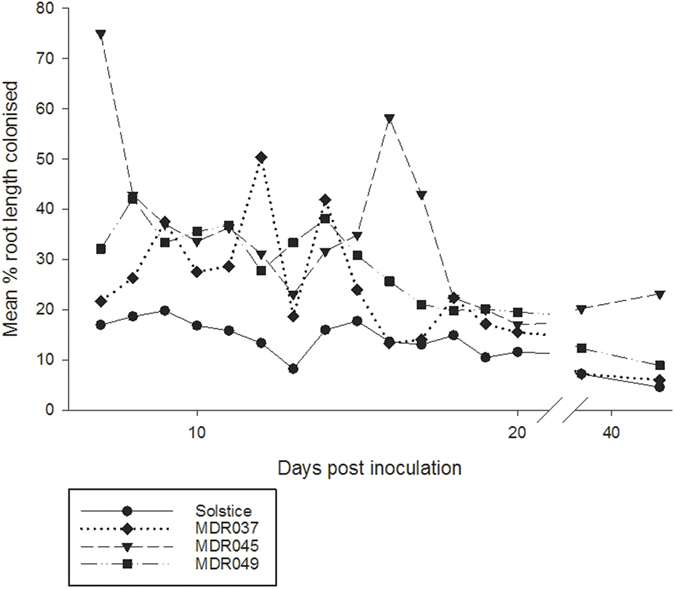
Percentage Root length colonisation (%RLC) of commercial mixed arbuscular mycorrhizas in *Triticum monococcum* varieties MDR037, MDR045 and MDR049 and *Triticum aestivum* variety Solstice from 7 to 48 days post inoculation. Standard error bars were removed to increase the graphs clarity.

Colonisation occurred rapidly and was successful in all varieties (Fig. 1). Over the first seven weeks of wheat growth, the percentage root length colonised (%RLC) of *T. monococcum* varieties MDR037, MDR045 and MDR049 decreased over time within the root system (*F*_15, 92_ = 2.574, *P* < 0.05). The *T. aestivum* variety Solstice showed a lower affinity towards the mycorrhizas than the *T. monococcum* varieties (*F*_3, 92_ = 11.049, *P* < 0.05). The %RLC of Solstice remained the same throughout the experiment despite the increasing root length.

Mycorrhizal quantification carried out after experiments showed that the %RLC was lower in the control plants than the mycorrhizal plants (*F*_*1, 247*_ = 15.87, *P* < 0.001) with the majority of control plants exhibiting no colonisation (Table 1).

### Aphid development and fecundity

**Figure 2:**
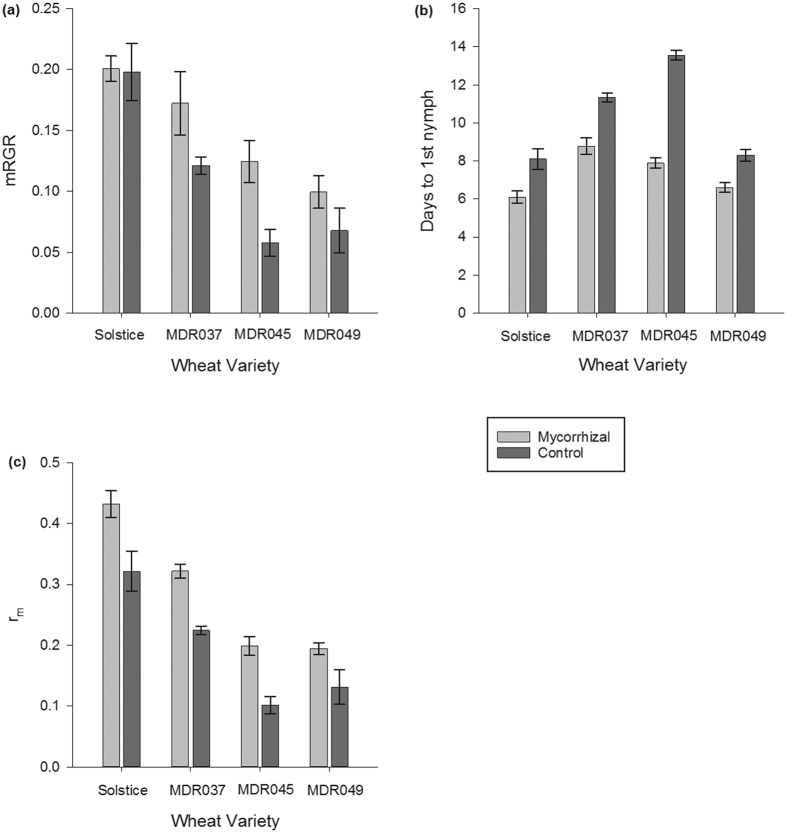
Development and reproductive success of *S. avenae* raised on mycorrhizal and control *Triticum monococcum* varieties MDR037, MDR045 and MDR049 and *Triticum aestivum* variety Solstice held within whole plant cages. (**a**) mRGR: mean relative growth rate. (**b**) Number of days to produce first nymph. (**c**) r_m_: intrinsic rate of increase. Error bars represent ± SEM.

The growth rate of *S. avenae* was lowest on MDR045 and MDR049 and highest on Solstice and MDR037 (*F*_3, 94_ = 25.797, *P* < 0.001), this was especially noticeable in the non-mycorrhizal plants (Fig. 2a). Colonisation by mycorrhizal fungi increased the growth rate of aphids raised on MDR037 and MDR045 plants (*F*_1, 94_ = 16.797, *P* < 0.001) (Fig. 2a). Furthermore, there was an interaction between the treatment and variety (*F*_3, 94_ = 3.912, *P* < 0.05) as mycorrhizas increased the growth rate of aphids on MDR037 and MDR045, but there was no effect on Solstice and MDR049.

There was a significant difference in the time aphids took to produce their first nymph. Aphids raised on MDR037 and MDR045 took the most number of days, whilst those raised on Solstice and MDR049 took the least number of days (*F*_3, 80_ = 48.636, *P* < 0.001) (Fig. 2b). Importantly, aphids on MDR049 and Solstice took the same number of days to produce their first nymph. Mycorrhizal colonisation greatly reduced the number of days before aphids produced their first nymph (*F*_1, 80_ = 48.636, *P* < 0.001) across all of the varieties (Fig. 2b). There was a strong interaction between the variety and treatment (*F*_3, 80_ = 4.606, *P* < 0.01) as AM fungi reduced the time aphids on MDR045 produced their first nymph to a much greater extent than the other three varieties.

Although there was no difference in the time aphids on MDR049 and Solstice took to produce their first nymph, this did not extend to the lifetime reproductive success as MDR045 and MDR049 had the lowest reproductive success and aphids raised on Solstice and MDR037 had the highest (*F*_3, 91_ = 52.153, *P* < 0.001) (Fig. 2c). The reproductive success was directly related to the growth rate results (Fig. 2a). The effects of AM fungi on the number of days to produce first nymph were similar to those observed for the reproductive success. Across all of the varieties, aphids on mycorrhizal plants had more reproductive success than those on non-mycorrhizal plants (*F*_1, 91_ = 29.790, *P* < 0.001) (Fig. 2c).

### Leaf anatomy

Vascular bundle width was greater in MDR037 plants than in MDR045 plants (*F*_1, 36_ = 14.68, *P* < 0.001) (Table 2). Mycorrhizal colonisation increased the bundle width consistently in both varieties (*F*_1, 36_ = 6.77, *P* < 0.05) (Table 2) so there was no interaction between the variety and the effect of colonisation.

### Aphid feeding behaviour

Overall, the addition of AM fungi had a large effect on the feeding behaviour which is apparent from the analysis of the feeding phases (Table 3).

### Probing (tissue penetration)

The duration of the first probe was on average 15 minutes longer in aphids feeding on MDR045 plants than MDR037 (*F*_1, 61_ = 4.590, *P* < 0.05). Mycorrhizal colonisation had no effect on the duration of the first probe.

### Pathway phase

The pathway (C) phase is defined as when the stylet is moving through the mesophyll towards the phloem. The number of pathway periods and the average time spent in the pathway phase did not differ between the varieties. Colonisation by mycorrhizas reduced the number of C phases that occurred (*F*_1, 61_ = 8.39, *P* < 0.01), but increased the average time spent within each C phase (*F*_1, 61_ = 6.62, *P* < 0.05); this was shown in both MDR037 and MDR045 varieties.

### Salivation and phloem feeding

Aphids on MDR037 exhibited more phloem feeding phases (E2) than those feeding on MDR045 (*F*_1, 61_ = 4.18, *P* < 0.05). The addition of AM fungi had no effect on the number of E2 phases. However, aphids on mycorrhizal plants fed on average for longer (*F*_1, 61_ = 5.28 *P* < 0.05) with a longer maximum phloem feeding period (*F*_1, 61_ = 4.333, *P* < 0.05). In both of these variables, the difference between the treatment and control was more pronounced in MDR045 than MDR037. Colonisation by AM fungi caused aphids on MDR045 to experience more sustained E2 phases, which lasted for longer than 10 minutes with the absence of salivation, (*F*_1, 61_ = 5.471, *P* < 0.05). The mycorrhizal fungal colonisation also caused aphids to spend a longer proportion of the overall time within the phloem (*F*_1, 61_ = 3.953, *P* ≤ 0.05).

### Penetration difficulties

There was no difference in the number of penetration difficulty events between aphids feeding on MDR037 and MDR045; however, aphids feeding on mycorrhizal plants of both varieties experienced approximately half the number of penetration difficulties than those feeding on control plants (*F*_1, 61_ = 16.71, *P* < 0.001).

### Alate aphid attraction and settlement

**Figure 3:**
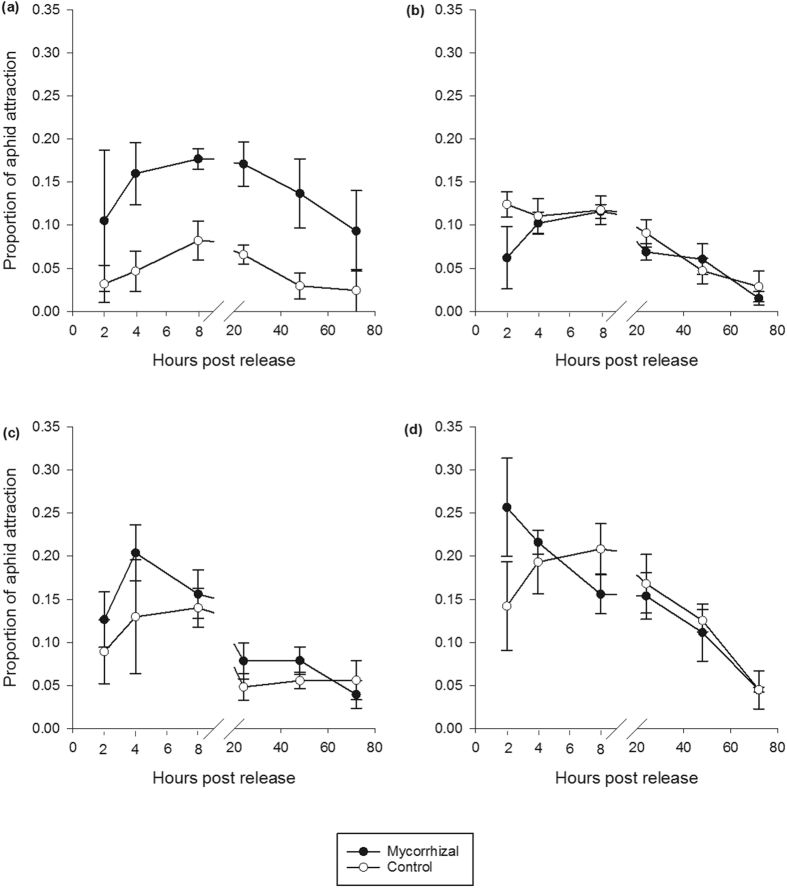
Attraction of alate aphids to mycorrhizal and control *Triticum monococcum* varieties and *Triticum aestivum* variety Solstice. (**a**) Solstice. (**b**) MDR037. (**c**) MDR045. (**d**) MDR049. Error bars represent ± SEM.

**Figure 4:**
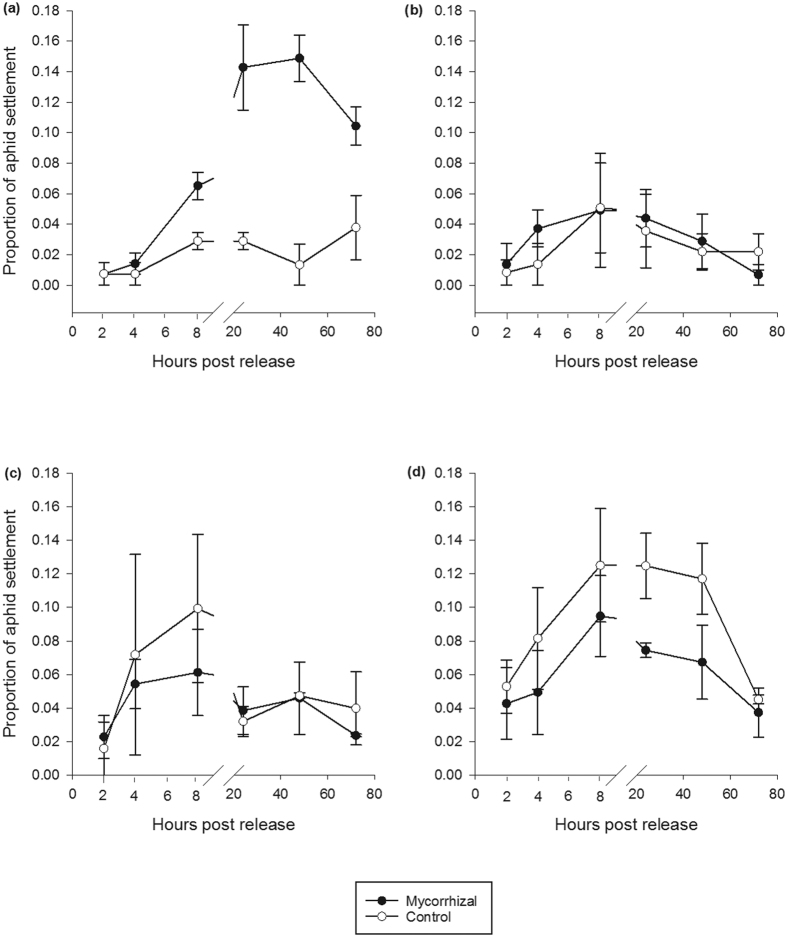
Alate aphid settlement on mycorrhizal and control *Triticum monococcum* varieties and *Triticum aestivum* variety Solstice. (**a**) Solstice. (**b**) MDR037. (**c**) MDR045. (**d**) MDR049. Error bars represent ± SEM.

Mycorrhizal colonisation led to more aphids settling on Solstice mycorrhizal plants. More alate aphids were attracted to the cage that held mycorrhizal Solstice plants (*F*_1, 12_ = 8.430, *P* < 0.05) (Fig. 3a) than control Solstice plants. Aphids also settled more on mycorrhizal Solstice plants; (*F*_1, 12_ = 23.882, *P* < 0.001) this only became apparent 8 hour post release (*F*_1, 8_ = 8.572, *P* ≤ 0.001) (Fig. 4a) showing an interaction between the treatment and hours post release (*F*_1, 12_ = 3.819, *P* < 0.05). The colonisation of mycorrhizal fungi had no effect on the attraction and settlement of aphids in the other varieties investigated (Figs 3 and 4).

## Discussion

We have combined EPG feeding behavioural analysis with studies on aphid reproductive success, pre- and post- alighting preferences, and leaf anatomy to unravel the effect of mycorrhizal colonisation on modern and ancestral wheat with different susceptibility to these destructive herbivores. Conflicting reports of the effects of AM fungi on plants are often based on a narrow range of experiments, but here we have shown that mycorrhizal colonisation differs between ancestral and modern wheat varieties, has a positive effect on aphid’s ability to phloem feed, induces susceptibility in a resistant wheat variety, and that this is likely associated with factors involving an increase in the size of the vascular bundle.

These results demonstrate the potential of AM fungi to affect plant resistance to aphids and induce susceptibility by interfering with the plants natural defence mechanism. The increased size of the sieve element could be indicative of a healthier and more nutritious plant. The higher level of salivation on resistant lines without AM fungi is indicative of problems with establishing phloem feeding, this suggests that the AM fungi are affecting the plants ability to block the sieve elements. Therefore, it is likely that there are multiple factors at play including plant anatomy, defence chemistry and plant health which we discuss herein.

Mycorrhizas can benefit their host plant by increasing their yield and nutrient acquisition^37^ especially those nutrients which are poor at diffusing through the soil such as P and Zn. Mycorrhizas do this by extending hyphae through the soil, thereby accessing these diffuse-poor nutrients^38^. Although mycorrhizas can colonise wheat varieties, the extent of symbiosis that occurs between the different cultivars and varieties is varied due to the ability of the fungus to absorb soil nutrients as well as the amount of carbon the plant will supply to the fungus^39^. Our work shows that there are differences in the mycorrhizal affinity depending on the wheat cultivar. The *T. aestivum* hexaploid susceptible variety Solstice was the most modern variety investigated in this study and it had the least affinity to the mycorrhizas. This is in keeping with previous experiments which showed that older crop varieties of current cultivars as well as ancestral cultivars have a higher level of symbiosis with AM fungi than more modern varieties^39^. The AM fungal colonisation in the *T. monococcum* varieties decreased over time, possibly due to the fungus proliferating at a slower rate than the root length increased, therefore creating a dilution effect. The AM fungi in the Solstice root system maintained a steady level of colonisation, suggesting that although Solstice has a lower affinity towards AM fungi, the fungi are able to proliferate more within the root system than in the *T. monococcum* varieties.

The multi-trophic interactions between mycorrhizas and herbivorous insects have been widely studied. The effect of mycorrhizal fungi on higher trophic levels is dependent on many biotic and abiotic factors, including soil phosphorus limitation as well as the variability that comes with different host species^26^. Here we have shown that the level of mycorrhizal colonisation is dependent on the *Triticum* species as *T. aestivum* var. Solstice had less AM fungi colonisation than *T. monococcum* varieties.

We investigated how the mycorrhizal colonisation affects aphid development and reproductive success. Results show variation within the diploid varieties and that the varieties MDR045 and MDR049 hold some resistance towards *S. avenae* with a slower aphid growth rate and a lower reproductive success. These results are in line with previous investigations of *T. monococcum* susceptibility to herbivorous insects. di Pietro *et al*.^23^ have shown that *S. avenae* aphids raised on *T. monococcum* varieties had lower reproductive success than on the susceptible *T. aestivum* variety Arminda. Arbuscular mycorrhizal fungal colonisation had less of an effect on aphids raised on Solstice as the AM fungi reduced the time to first nymph being produced and their reproductive success, but had no effect on growth rate. Solstice had the least affinity towards the commercial mycorrhizas so the effect of the treatment may be too low to be noticed in these experiments. Mycorrhizal root colonisation increased the growth rate of aphids on MDR037 and MDR045, as well as reproductive success of aphids on MDR037, MDR045 and MDR049. Mycorrhizal colonisation is known to increase life history traits of aphids including growth rate and reproductive success^27^. These findings are in keeping with Koricheva *et al*.^25^ showing that AM fungi affects *S. avenae* on ancestral plants in the same way as other phloem feeders on modern plants. To date, the mechanism of this interaction was unknown; it could be due to many factors including increased leaf size^40^, plant height and biomass (Supplementary material Fig. 1) in which there would be more area for potential feeding. However, we have shown that the internal anatomy of the leaf can be influenced by mycorrhizas, and factors related to this are likely to be a significant reason why aphid feeding success (and thence reproduction) is greater on mycorrhizal plants. Aphid sap ingestion takes place through phloem sieve elements which are transport channels of vascular bundles^41^ and one previous study has also shown that mycorrhizas can increase the size of vascular bundles^35^. This could structurally aid aphid sap ingestion, by providing a larger target and thus increased chance of phloem location, resulting in increased susceptibility to attack^42^. The vascular bundle width of MDR037 was greater than MDR045, and AM fungi caused an increasein bundle width of both MDR037 and MDR045. The behavioural studies complement this, showing that aphids on mycorrhizal plants spent more time ingesting phloem and experienced longer sustained ingestion events. The effect of AM fungi on vascular bundles and other physical defence mechanisms including the role of callose and proteins^43^ on aphid behaviour would need to be studied in future experiments. Therefore, we make the link between aphid feeding behaviour and plant vascular bundle size; which are both positively influenced by mycorrhizal colonisation.

Our EPG results support other studies where aphids are able to locate the phloem on resistant varieties, but with reduced phloem ingestion or feeding periods and increased salivation^22^,^44^. These results suggest that there could be further aspects of resistance other than leaf anatomy, as they occurred in the absence of mycorrhizas. A number of possible mechanisms have been suggested, such as; the sap is unable to flow easily within the phloem due to blocked sieve elements^41^, the nutritional quality of the sap is altered by differences in the amino acid, sucrose and sugar: amino acid ratio^45^ or that the wound created in the phloem alerts the plant defences faster^45^. It is unlikely, however, that a nutritional change in the sap is key to the resistance as studies investigating sap quality^46^,^47^ show that it is only loosely linked to aphid feeding and that there are other factors involved in aphid feeding behaviour.

Metabolites may also play a role in resistance; Greenslade *et al*.^22^ showed distinct metabolic phenotypes for resistant *T. monococcum* lines in comparison to susceptible lines. The expression of trehalose was shown to be elevated in *T. monococcum* MDR049 and MDR657 and *T. aestivum* Solstice after aphid infestation^22^. The disaccharide sugar trehalose has been shown to increase plant resistance to the Powdery mildew pathogen (*Blumeria graminis*) by accelerating the stress signalling response^48^. During the probing phase, the aphids salivate which aids the stylet as it passes through the mesophyll, and that the saliva will prevent the plant responding to the phloem wound created as well as preventing the sieve elements from blocking^47^. The germination of AM fungal spores has been shown to decrease trehalose^49^ and mycorrhizas have been shown to increase the enzyme trehalase activity in plant leaves and roots^50^. Trehalase acts independently from trehalose^51^ breaking its substrate down into two glucose molecules^52^. The feeding behaviour investigations showed that aphids were able to feed for longer on the mycorrhizal plants. The higher activity of trehalase in mycorrhizal plants would reduce the activity of trehalose and in turn the stress signalling response would be slower in the mycorrhizal plants, allowing the aphid to feed more. Further investigations are needed to determine whether the addition of mycorrhizal fungi increases trehalase and decreases trehalose within *T. monococcum* leaves and if there is any associated effect on aphid feeding behaviour.

Mycorrhizas can manipulate the emissions of VOC’s from plants which can increase the attraction of the plant to insects^28^,^53^. Our results showed that the addition of AM fungi only affected the attraction of aphids to *T. aestivum* variety Solstice, with alate *S. avenae* being attracted to and settling more on the mycorrhizal plants. Aphids on these plants had increased reproductive success, but there was no effect on the growth rate. We would expect this to translate into increased aphid infestation on mycorrhizal compared to non-mycorrhizal plants of this variety. Therefore, it is encouraging that Solstice had the lowest affinity towards AM fungi in the varieties studied. Babikova *et al*.^28^ showed that AM fungi inhibited insect repellent VOC sequiterpenes and these are known to be present in the volatile profile of *T. aestivum*^54^. To our knowledge, the levels of sesquiterpenes in ancestral *Triticum* varieties and to what level AM fungi can affect them are unknown. This would be a most useful avenue for future research.

In summary, we conclude that AM fungi have a higher affinity to ancestral than modern wheat varieties and that any associated benefit to the plant can be compromised due to associated effects on the plant defences against aphids. The ability to alter the resistance level of a plant through the addition of AM fungi opens up avenues for understanding the mechanisms underlying plant resistance to aphids, and aphid-plant interactions, which have long eluded researchers.

## Materials and Methods

### Plant and insect materials

An anholocyclic *S. avenae* culture originally collected from Rothamsted Research, Harpenden, Hertfordshire was used for all bioassay experiments and reared on *Triticum aestivum* variety Tybalt at 22 °C (±2 °C), 16:8 h light: dark photoperiod and 43% humidity.

*Triticum monococcum* lines MDR037, MDR045 and MDR049 and *Triticum aestivum* variety Solstice were provided by Rothamsted Research. Seeds were stored at 4 °C whilst plants were kept in a controlled environment room at 22 °C (±2 °C), 16:8 h light: dark photoperiod and 43% humidity to prevent production of sexual forms and watered approximately with 8 ml daily. Plants were seven days old at the start of all bioassays.

The commercial AM fungal product “Rootgrow” was provided by PlantWorks (Sittingbourne, Kent) and stored at 4 °C. Rootgrow is made up of a mixture of arbuscular mycorrhizal fungi which are of UK origin.

Soil used for growing all plants was provided by Petersfield Products (Cosby, Leicester). The soil composition was 75% medium grade peat, 12% screened sterilised loam, 3% medium grade vermiculite and 10% grit. The N content of the soil was 14% and the P_2_O_5_ content was 16%.

### Arbuscular Mycorrhiza inoculation and detection

Inoculation occurred at the time of sowing seeds; pots of 4 cm diameter were filled with approximately 40 g of soil. Then 2.5 ml of inoculum was mixed in with the top layer of soil, the seed was added on top of this inoculum mixed soil and approximately 3 g soil added on top of the seed. The control was treated identically but used inoculum that had been autoclaved and allowed to dry for 12 h before sowing. Elimination of bacteria in the blending process of the inoculum meant that a microbial filtrate was not required in control treatments. At the end of each experiment (see below), the roots were extracted from the soil and stored in 70% ethanol at room temperature until they were stained for visualisation. The AM fungi visualisation method followed that of Vierheilig *et al*.^55^, root clearing was carried out in 10% potassium hydroxide in a water bath at 65 °C, and the time for the roots to clear was changed to 4 minutes. After this, the roots were rinsed with tap water and placed in a staining solution (84.4:15:0.6, dH_2_O: 1%HCl: Quink) in a water bath at 75 °C for 15 minutes. Stained roots were mounted onto microscope slides with coverslips. AM fungi root structures were quantified by the cross-hair eye piece method^56^ at 200 x magnification.

### Aphid development and fecundity

Development and fecundity bioassays were done on all four lines in a controlled environment room (22 °C (±2 °C), 16:8 h L:D). Plants were watered with approximately 8 ml daily. The pots were placed in a randomised block design with 10 replicates per variety and treatment.

Three mature apterous aphids were placed on each plant within a whole plant cage which consisted of insect proof netting with a plastic support structure. These mature aphids were allowed to larviposit overnight. The following morning the mature aphids were removed and the number of nymphs produced recorded. The neonate nymphs (<1 day old) were weighed and transferred back to the plant of the same treatment and variety and left undisturbed for seven days. After seven days, the number of survivors was recorded and survivors re-weighed to determine the mean Relative Growth Rate (mRGR). This was calculated^57^,^58^ as:


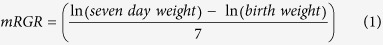


After re-weighing, one of the nymphs was chosen at random and transferred back to their original plant. These aphids were left undisturbed to develop and were monitored every day; all of the aphids developed into apterous aphids. The time taken to produce their first nymph (FD) and the number of nymphs they produced over their lifetime (D) were recorded to calculate the intrinsic rate of increase using a constant from the mean pre-reproductive time for aphid species^61^. Subsequent nymphs were removed to prevent overcrowding. The intrinsic rate of increase was calculated as:


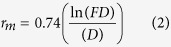


### Aphid host choice

Alate *S. avenae* were produced by crowding apterous aphids on *T. aestivum* variety Tybalt. Solstice, MDR037, MDR045 and MDR049 control and mycorrhizal treated plants were sown in small pots of 4 cm diameter, as above. There were 10 replicates for each plant and treatment. The structure used for this experiment consisted of two 30 cm^3^ cages linked by a 60 cm × 15 cm tunnel. Each trial used one plant variety at a time with ten control plants in one cage and ten mycorrhizal plants placed in the other cage. Across the three experimental replicates, the cage which had the control and the cage which had treatment plants were switched to determine if the experimental design played a role. Between forty and fifty alate aphids were released in the middle of the tunnel each time. The number of aphids that were within each cage and the number of aphids that settled on each plant were recorded at 2, 4, 8, 24, 48 and 72 hours after release. Attraction was defined as aphids which were within the cage; settlement was defined as aphids on plants. The proportion of aphids attracted to and settled on the mycorrhizal and control plants was recorded.

### Leaf anatomy

Mature leaves from 7 day old *T. monococcum* MDR037 and MDR045 were selected at random. Each leaf was fixed in a mixture of formalin, ethyl alcohol and acetic acid mixture and embedded in paraffin wax, as described by El-Afry *et al*.^59^. Sections were taken by rotary microtome to a thickness of 8 μm and stained with safranine and light green. Sections were examined under light microscopy and width of vascular bundles measured, using an eye piece graticule. Ten replicate mycorrhizal and non-mycorrhizal plants of each variety were used.

### Electrical Penetration Graph recording of aphid feeding behaviour

The Direct Current Electrical Penetration Graph (EPG) method^36^ was used to investigate the feeding behaviour of *S. avenae* on mycorrhizal and control *T. monococcum* MDR037 and MDR045. Apterous *S. avenae* were collected from *T. aestivum* cv Tybalt and starved for 1.5 h before attaching 20 μm gold wire of 1.5–2 cm length to the dorsum of the aphid using water based adhesive containing silver paint. This was also used to connect the gold wire to a 3 cm copper wire which in turn was connected to a brass pin. The brass pin was connected to an EPG probe which was connected to an 8 channel Giga 8 Direct Current amplifier (EPG systems, Wageningen, The Netherlands). A ground electrode was inserted into the soil of each of the potted plants and connected to the amplifier. This experimental set-up was encaged within a grounded Faraday cage. Each aphid was placed on the first leaf of an individual seven-day old *T. monococcum* plant which was placed upon an upside down 100 ml beaker. A petri dish filled with water was placed underneath each potted plant to ensure that the soil stayed moist for electrical conductivity. The recordings were collected continuously for 8 hours using Stylet + data acquisition software (EPG systems, Wageningen, The Netherlands). During recordings, plants were kept at room temperature and under constant light. Two replicates of each of the varieties and treatments were carried out each day with the plants placed in a randomised order. A total of 104 aphids were investigated.

### Data processing of EPG aphid feeding behaviour experiments

The waveform patterns were identified, interpreted and annotated using Stylet + analysis software (EPG systems, Wageningen, The Netherlands) to correlate waveforms to aphid behaviour. The waveforms were placed into the following categories: non-probing, phloem sieve element salivation (E1), phloem sieve element ingestion (E2), penetration difficulties (F), xylem drinking (G) and stylet pathway phase (C)^36^,^60^. Responses were valid if a waveform was present within the first hour, and if waveform activity lasted for at least 30 minutes in the last hour of recordings. Annotated results were then imported into EPG analysis Microsoft Excel macro (EPG systems, Wageningen, The Netherlands) which calculated the variables from the annotated feeding behaviour.

### Data analysis

The mycorrhizal colonisation of the different wheat varieties was compared with a one-factor analysis of variance in which the variety of *Triticum* was the independent factor. Data were subject to the angular transformation before analysis. Mycorrhizal colonisation of control and treatment plant roots after the completion of each experiment were compared using a two sample student’s T-Test with a group factor. Data were subject to square root transformation before analysis.

The differences in aphid mean relative growth rate (mRGR), average number of days to produce their first nymph and the intrinsic rate of increase (r_m_) were examined with a two-factor analysis of variance (ANOVA) with the wheat variety and the mycorrhizal treatment as factors. The development data were subject to square root transformation.

The effect of mycorrhizal treatment on both the attraction and settlement of aphids over the course of 72 hours were determined by carrying out a multiple regression analysis in which the hours post release and the treatment were the independent factors.

The effect of mycorrhizal colonisation on vascular bundle width was analysed using a two-factor ANOVA with the variety and mycorrhizal treatment being the factors.

All of these comparative analyses were carried out in SPSS Statistics ^®^ for Windows (2012, 21^st^ Edition, © IBM Corp, Armonk, New York, USA).

Electrical Penetration Graph results were analysed by a linear mixed model fitted by restricted maximum likelihood (REML) in Genstat ^®^ (2013, 16^th^ Edition, © VSN International Ltd, Hemel Hempstead, UK). Due to the validity process the number of replicates was uneven across varieties and treatment; MDR037 control n = 15, MDR037 treatment n = 12, MDR045 control n = 16 and MDR045 treatment n = 19. Each of the 37 variables recorded were analysed separately and where necessary, the variable was subject to log, square root or logit transformations so the results conformed to the assumed variance homogeneity and residual normality. Variables that contained values of zero were off-set by adding half of the lowest value above zero that was recorded.

## Additional Information

**How to cite this article:** Simon, A. L. *et al*. Unravelling mycorrhiza-induced wheat susceptibility to the English grain aphid *Sitobion avenae. Sci. Rep.* **7**, 46497; doi: 10.1038/srep46497 (2017).

**Publisher's note:** Springer Nature remains neutral with regard to jurisdictional claims in published maps and institutional affiliations.

## Supplementary Material

Supplementary Material

## Figures and Tables

**Table 1:** Mean Percentage Root length colonisation (%RLC) (±SE) of mycorrhizal and control *Triticum monococcum* varieties MDR037, MDR045 and MDR049 and *Triticum aestivum* variety Solstice.

Solstice		MDR037		MDR045		MDR049	
Control	Mycorrhizal	Control	Mycorrhizal	Control	Mycorrhizal	Control	Mycorrhizal
0.367 ± 0.248	17.902 ± 1.931	0.088 ± 0.044	26.507 ± 4.145	0.269 ± 0.101	26.719 ± 4.221	0.053 ± 0.031	41.655 ± 6.785

Quantification was carried out after other experiments were completed.

**Table 2:** Mean vascular bundle width (±SE) of aphid susceptible (MDR037) and resistant (MDR045) *T. monococcum* varieties.

MDR037		MDR045	
Control	Mycorrhizal	Control	Mycorrhizal
83.2 ± 1.09	85.8 ± 0.59	79 ± 1.14	81.9 ± 1.26

**Table 3:** Feeding behaviour of *Sitobion avenae* feeding on mycorrhizal treated and control *Triticum monococcum* varieties MDR037 and MDR045.

Variables	MDR037		MDR045	
	Control	Mycorrhizal	Control	Mycorrhizal
Qualifying replicates	12	15	19	16
**Probing (tissue penetration):**				
Number of probes	8.467 ± 1.044	10.923 ± 0.727	9.471 ± 0.910	8.15 ± 1.317
Duration of 1st probe	30.658 ± 19.294	18.770 ± 4.742	26.153 ± 11.514	47.308 ± 17.567
**Pathway:**				
Number of pathway periods (C)	19 ± 2.3	18 ± 1.7	24 ± 2.1	15 ± 1.5
Average time in pathway (C)	9.119 ± 0.468	10.542 ± 0.336	7.865 ± 0.585	9.214 ± 0.428
**Salivation and phloem feeding:**				
Time to 1st phloem feeding (E2) from 1st salivation (E1)	152.417 ± 25.186	179.567 ± 26.329	269.557 ± 25.885	161.340 ± 20.312
Number of phloem feeding events (E2)	3.2 ± 0.21	2.46 ± 0.34	1.18 ± 0.58	2 ± 0.51
Average time of phloem feeding event (E2)	14.595 ± 11.371	24.069 ± 7.985	8.031 ± 1.243	56.330 ± 11.705
Maximum time of a phloem feeding event (E2)	39.523 ± 23.274	38.690 ± 16.829	12.911 ± 2	95.593 ± 22.165
Number of sustained phloem events (sE2)	0.8 ± 0.137	0.692 ± 0.176	0.294 ± 0.098	0.7 ± 0.142
% time ingestion	9.668 ± 3.556	7.279 ± 3.233	1.616 ± 0.920	12.862 ± 2.694
**Stylet penetration difficulties:**				
Number of stylet penetration difficulties (F)	5.7 ± 1	2.1 ± 0.7	9.8 ± 1	3.2 ± 0.4

Mean (±SE) EPG variables; total duration (in seconds), frequency and average duration (predicted means) from 8 hour recordings of feeding.

## References

[b1] Dixon A. F. G. Aphid ecology. An optimization approach. (Chapman and Hall, London, 1998).

[b2] Blackman R. L. & Eastop V. F. Aphids on the World’s Herbaceous Plants and Shrubs. (John Wiley & Sons Ltd, 2007).

[b3] Tagu D., Klingler J. P., Moya A. & Simon J. C. Early progress in aphid genomics and consequences for plant-aphid interactions studies. Mol. Plant-Microbe Interact. 21, 701–708 (2008). 18624634 10.1094/MPMI-21-6-0701

[b4] Agrawal A. A. Macroevolution of plant defense strategies. Trends Ecol. Evol. 22, 103–109 (2007). 17097760 10.1016/j.tree.2006.10.012

[b5] Rasmann S. & Agrawal A. A. Plant defense against herbivory: progress in identifying synergism, redundancy, and antagonism between resistance traits. Curr. Opin. Plant Biol. 12, 473–478 (2009). 19540153 10.1016/j.pbi.2009.05.005

[b6] Thaler J. S. Jasmonate-inducible plant defences cause increased parasitism of herbivores. Nature 399, 686–688 (1999).

[b7] Rasmann S., Chassin E., Bilat J., Glauser G. & Reymond P. Trade-off between constitutive and inducible resistance against herbivores is only partially explained by gene expression and glucosinolate production. J. Exp. Bot. 66, 2527–2534 (2015). 25716695 10.1093/jxb/erv033 PMC4986863

[b8] Turley N. E. & Johnson M. T. J. Ecological effects of aphid abundance, genotypic variation, and contemporary evolution on plants. Oecologia 178, 747–759 (2015). 25740334 10.1007/s00442-015-3276-8

[b9] Züst T. & Agrawal A. A. Mechanisms and evolution of plant resistance to aphids. Nat. Plants 2, 1–9 (2016). 10.1038/nplants.2015.206 27250753

[b10] Czesak M. E., Fritz R. S. & Hochwender C. Selection and Genetic Architecture of Plant Resistance in Specialization, Speciation and Radiation. The Evolutionary Biology of Herbivorous Insects.(2008).

[b11] Hartley S. E. & Gange A. C. Impacts of plant symbiotic fungi on insect herbivores: mutualism in a multitrophic context. Ann. Rev. Entomol. 54, 323–342 (2009). 19067635 10.1146/annurev.ento.54.110807.090614

[b12] VanDoorn A. & de Vos M. Resistance to sap-sucking insects in modern-day agriculture. Front. Plant Sci. 4, 222: doi: 210.3389/fpls.2013.00222 (2013). 23818892 10.3389/fpls.2013.00222 PMC3694213

[b13] Foster S. P. . A mutation (L1014F) in the voltage-gated sodium channel of the grain aphid, *Sitobion avenae*, is associated with resistance to pyrethroid insecticides. Pest Manag. Sci. 70, 1249–1253 (2014). 24227679 10.1002/ps.3683

[b14] Carvalho F. P. Agriculture, pesticides, food security and food safety. Environ. Sci. Policy 9, 685–692 (2006).

[b15] Shewry P. R. Wheat. J. Exp. Bot. 60, 1537–1553 (2009). 19386614 10.1093/jxb/erp058

[b16] Shewry P. R. & Hey S. J. The contribution of wheat to human diet and health. Food Energy Secur. 4, 178–202 (2015). 27610232 10.1002/fes3.64 PMC4998136

[b17] Fiebig M., Poehling H. M. & Borgemeister C. Barley yellow dwarf virus, wheat, and *Sitobion avenae*: A case of trilateral interactions. Entomol. Exp. Appl. 110, 11–21 (2004).

[b18] Emden H. F. & Harrington R. Aphids as Crop Pests. (CABI, 2007).

[b19] Sotherton N. W. & Lee G. Field assessments of resistance to the aphids *Sitobion avenae* and *Metopolophium dirhodum* in old and modern spring-sown wheats. Ann. Appl. Biol. 112, 239–248 (1988).

[b20] Salamini F., Ozkan H., Brandolini A., Schäfer-Pregl R. & Martin W. Genetics and geography of wild cereal domestication in the near east. Nat. Rev. Genet. 3, 429–441 (2002). 12042770 10.1038/nrg817

[b21] Elek H. . The effect of the A genome species (*Triticum monococcum and Triticum boeoticum*) on the fecundity and behaviour of *Rhopalosiphum padi* - bird cherry-oat aphid. Georg. Agric. 15, 1–17 (2012).

[b22] Greenslade A. F. C. . *Triticum monococcum* lines with distinct metabolic phenotypes and phloem based resistance to the bird cherry oat aphid *Rhopalosiphum padi*. Ann. Appl. Biol. 168, 435–449 (2016). 27570248 10.1111/aab.12274 PMC4982108

[b23] Di Pietro J. P., Caillaud C. M., Chaubet B., Pierre J. S. & Trottet M. Variation in Resistance to the Grain Aphid, *Sitobion avenae* (Sternorhynca: Aphididae), Among Diploid Wheat Genotypes: Multivariate Analysis of Agronomic Data. Plant Breed. 117, 407–413 (1998).

[b24] Brundrett M. Mycorrhizas in Natural Ecosystems. Adv. Ecol. Res. 21, 171–313 (1991).

[b25] Koricheva J., Gange A. C. & Jones T. Effects of mycorrhizal fungi on insect herbivores: A meta-analysis. Ecology 90, 2088–2097 (2009). 19739371 10.1890/08-1555.1

[b26] Hoeksema J. D. . A meta-analysis of context-dependency in plant response to inoculation with mycorrhizal fungi. Ecol. Lett. 13, 394–407 (2010). 20100237 10.1111/j.1461-0248.2009.01430.x

[b27] Gange A. C., Bower E. & Brown V. K. Positive effects of an arbuscular mycorrhizal fungus on aphid life history traits. Oecologia 120, 123–131 (1999). 28308043 10.1007/s004420050840

[b28] Babikova Z. . Arbuscular mycorrhizal fungi and aphids interact by changing host plant quality and volatile emission. Funct. Ecol. 28, 375–385 (2014).

[b29] Hause B., Maier W., Miersch O., Kramell R. & Strack D. Induction of jasmonate biosynthesis in arbuscular mycorrhizal barley roots. Plant Physiol. 130, 1213–1220 (2002). 12427988 10.1104/pp.006007 PMC166642

[b30] Hempel S. . Specific bottom-up effects of arbuscular mycorrhizal fungi across a plant-herbivore-parasitoid system. Oecologia 160, 267–277 (2009). 19219458 10.1007/s00442-009-1294-0 PMC2757589

[b31] Abdelkarim B., Ownley B. H., Klingeman W. E. & Gwinn K. D. Effect of arbuscular mycorrhizae on aphid infestation of wheat. Phytopathology 101, S2 (2011).

[b32] Williams A., Birkhofer K. & Hedlund K. Above- and below-ground interactions with agricultural management: Effects of soil microbial communities on barley and aphids. Pedobiologia 57, 67–74 (2014).

[b33] Gange A. C. & West H. M. Interactions between arbuscular mycorrhizal fungi and foliar-feeding insects in *Plantago lanceolata* L. New Phytol. 128, 79–87 (1994). 10.1111/j.1469-8137.1994.tb03989.x 33874534

[b34] Grabmaier A., Heigl F., Eisenhauer N., van der Heijden M. G. A. & Zaller J. G. Stable isotope labelling of earthworms can help deciphering belowground-aboveground interactions involving earthworms, mycorrhizal fungi, plants and aphids. Pedobiologia 57, 197–203 (2014).

[b35] Krishna B. Y. K. R., Suresh H. M. & Sunder J. S. Y. A. M. Changes in the Leaves of finger millet due to VA mycorrhizal Infection. New Phytol. 87, 717–722 (1981).

[b36] Tjallingii W. F. & Hogen Esch T. Fine structure of aphid stylet routes in plant tissues in correlation with EPG signals. Physiol. Entomol. 18, 317–328 (1993).

[b37] Lewis J. & Koide R. Phosphorus supply, mycorrhizal infection and plant offspring vigour. Funct. Ecol. 4, 695–702 (1990).

[b38] Harley J. L. & Smith S. E. Mycorrhizal Symbiosis. (Academic Press, 1983).

[b39] Hetrick B. A. D., Wilson G. W. T. & Cox T. S. Mycorrhizal dependence of modern wheat cultivars and ancestors: a synthesis. Can. J. Bot. 71, 512–518 (1993).

[b40] Smith S. E. & Read D. J. Mycorrhizal symbiosis. (Academic Press, 1997).

[b41] Van Bel A. J. E. The phloem, a miracle of ingenuity. Plant, Cell Environ. 26, 125–149 (2003).

[b42] Aravind M. B. & Kajjidoni S. T. Leaf anatomical basis of woolly aphid resistance in sugarcane. Curr. Sci. 93, 906–909 (2007).

[b43] Will T., Furch A. C. U. & Zimmermann M. R. How phloem-feeding insects face the challenge of phloem-located defenses. Front. Plant Sci. 4, 336 (2013). 24009620 10.3389/fpls.2013.00336 PMC3756233

[b44] Caillaud C. M., di Pietro J. P., Chaubet B. & Pierre J. S. Application of discriminant-analysis to electrical penetration graphs of the aphid *Sitobion avenae* feeding on resistant and susceptible wheat. J. Appl. Entomol. 119, 103–106 (1995).

[b45] Dinant S., Bonnemain J. L., Girousse C. & Kehr J. Phloem sap intricacy and interplay with aphid feeding. Comptes Rendus - Biol. 333, 504–515 (2010). 10.1016/j.crvi.2010.03.008 20541162

[b46] Sandstrom J. Nutritional quality of phloem sap in relation to host plant-alternation in the bird cherry-oat aphid. Chemoecology 10, 17–24 (2000).

[b47] Sagers C. L. & Goggin F. L. Isotopic enrichment in a phloem-feeding insect: Influences of nutrient and water availability. Oecologia 151, 464–472 (2007). 17124569 10.1007/s00442-006-0603-0

[b48] Reignault P. . Trehalose induces resistance to powdery mildew in wheat. New Phytol. 149, 519–529 (2002). 10.1046/j.1469-8137.2001.00035.x 33873340

[b49] Bécard G., Doner L. W., Rolin D. B., Douds D. D. & Pfeffer P. E. Identification and quantification of trehalose in vesicular-arbuscular mycorrhizal fungi by *in vivo* 13C NMR and HPLC analyses. New Phytol. 118, 547–552 (1991).

[b50] Schellenbaum L., Müller J. & Boller T., Wiemken, a. & Schüepp, H. Effects of drought on non-mycorrhizal and mycorrhizal maize: Changes in the pools of non-structural carbohydrates, in the activities of invertase and trehalase, and in the pools of amino acids and imino acids. New Phytol. 138, 59–66 (1998).

[b51] Wisser G., Guttenberger M., Hampp R. & Nehls U. Identification and characterization of an extracellular acid trehalase from the ectomycorrhizal fungus Amanita muscaria. New Phytol. 146, 169–175 (2000).

[b52] Becker A., Schlöder P., Steele J. E. & Wegener G. The regulation of trehalose metabolism in insects. Experientia 52, 433–439 (1996). 8706810 10.1007/BF01919312

[b53] Schausberger P., Peneder S., Jürschik S. & Hoffmann D. Mycorrhiza changes plant volatiles to attract spider mite enemies. Funct. Ecol. 26, 441–449 (2012).

[b54] Kong C. H., Wang P. & Xu X. H. Allelopathic interference of Ambrosia trifida with wheat (Triticum aestivum). Agric. Ecosyst. Environ. 119, 416–420 (2007).

[b55] Vierheilig H., Coughlan A. P., Wyss U. R. S. & Recherche C. De. Ink and Vinegar, a Simple Staining Technique for Arbuscular-Mycorrhizal Fungi. Appl. Environ. Microbiol. 64, 5004–5007 (1998). 9835596 10.1128/aem.64.12.5004-5007.1998 PMC90956

[b56] McGonigle T. P., Miller M. H., Evans D. G., Fairchild G. L. & Swan J. A. A new method which gives an objective measure of colonization of roots by vesicular—arbuscular mycorrhizal fungi. New Phytol. 115, 495–501 (1990). 10.1111/j.1469-8137.1990.tb00476.x 33874272

[b57] Radford P. J. Growth Analysis Formulae - Their Use and Abuse. Crop Sci. 7, 171–175 (1967).

[b58] Leather S. R. & Dixon A. F. G. Aphid growth and reproductive rates. Entomol. Exp. Appl. 35, 137–140 (1984).

[b59] Wyatt I. J. & White P. F. Simple estimation of intrinsic increase rates for aphids and tetranychid mites. J. Appl. Ecol. 14, 757–766 (1977).

[b60] El-Afry M. M., El-Nady M. F., Abdelmonteleb E. B. & Metwaly M. M. S. Anatomical studies on drought-stressed wheat plants (*triticum aestivum* l.) treated with some bacterial strains. Acta Biol. Szeged. 56, 165–174 (2012).

[b61] Pettersson J., Tjallingii W. F. & Hardie J. Host-plant selection and feeding. In Aphids as Crop Pests.(CAB International, 2007).

