# Supplementary material for: Unravelling mycorrhiza-induced wheat susceptibility to the English grain aphid Sitobion avenae
Source: Sci Rep. 2017 Apr 13;7:46497. doi: 10.1038/srep46497 (PMC5390270; doi:10.1038/srep46497)
Supplement: Supplementary Material [file srep46497-s1.pdf]

Unravelling mycorrhiza-induced wheat susceptibility to the English grain aphid *Sitobion avenae*

Amma L. Simon[a,c], Peter A.D. Wellham[b], Gudbjorg I. Aradottir[a] and Alan C. Gange[c]

[a]*Department of Biological Chemistry and Crop Protection, Rothamsted Research, Harpenden, Hertfordshire, AL5 2JQ, UK.*

[b]*Somerville College, University of Oxford, Oxford, OX2 5HD, UK.*

[c]*School of Biological Sciences, Royal Holloway University of London, Egham, Surrey, TW20 0EX, UK.*

Corresponding author: amma.simon@rothamsted.ac.uk

Supplementary material

Life history traits of T. aestivum and T. monococcum inoculated with varying amounts of mycorrhizas

Plants were inoculated in the same fashion as previous assays, 2.5, 5 and 10ml of inoculum was mixed in with the top layer of soil. Plant height at 7days and 12days was measured. Fourteen day old plants were uprooted from the soil, the roots and the seeds were removed. Each plant was weighed to determine the wet biomass. After weighing, each plant was inserted into a paper bag with 8-10 holes to provide ventilation; these were placed in an oven at 60°C for 6-8 hours before weighing to determine the dry biomass weight.

Figure 1. Life history traits of *Triticum monococcum* varieties MDR037, MDR045 and MDR049 and *Triticum aestivum* var. Solstice grown in soil with the addition of 2.5, 5 and 10ml of mycorrhizas. (**a**) Plant height of 7 day old plants. (**b**) Plant height of 12 day old plants. (**c**) Wet biomass of 14 day old plants. (**d**) Dry biomass of 14 day old plants.

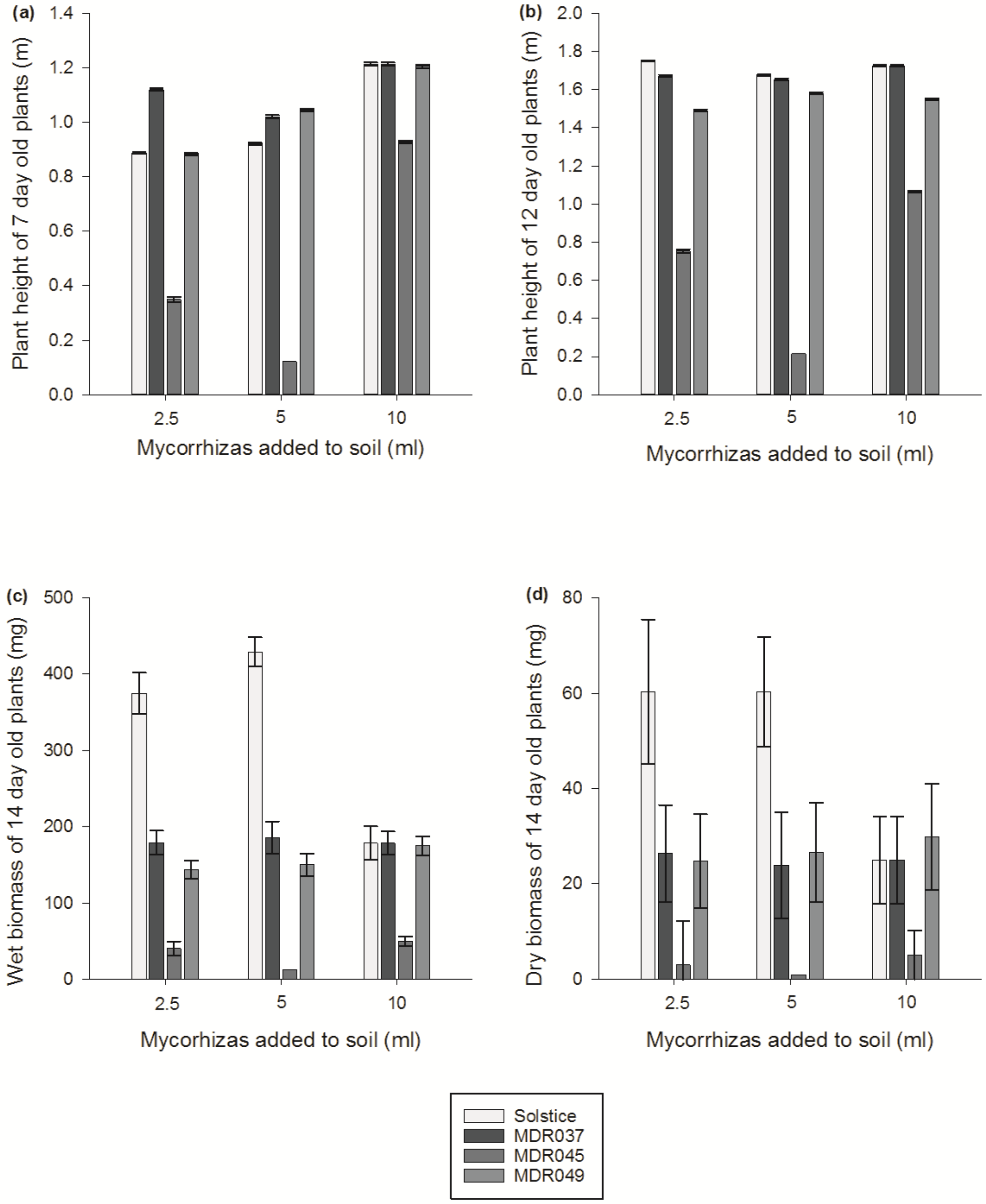

(a)
Plant height of 7 day old plants (m)
1.4
1.2
1.0
0.8
0.6
0.4
0.2
0.0
2.5
5
10
Mycorrhizas added to soil (ml)
(b)
Plant height of 12 day old plants (m)
2.0
1.8
1.6
1.4
1.2
1.0
0.8
0.6
0.4
0.2
0.0
2.5
5
10
Mycorrhizas added to soil (ml)
(c)
Wet biomass of 14 day old plants (mg)
500
400
300
200
100
0
2.5
5
10
Mycorrhizas added to soil (ml)
(d)
Dry biomass of 14 day old plants (mg)
80
60
40
20
0
2.5
5
10
Mycorrhizas added to soil (ml)
Solstice
MDR037
MDR045
MDR049
